# The paediatric flat foot and general anthropometry in 140 Australian school children aged 7 - 10 years

**DOI:** 10.1186/1757-1146-4-12

**Published:** 2011-04-22

**Authors:** Angela M Evans

**Affiliations:** 1 School of Health Science, Division of Health Science, University of South Australia, City East Campus, North Terrace, Adelaide 5000, South Australia

## Abstract

**Background:**

Many studies have found a positive relationship between increased body weight and flat foot posture in children.

**Methods:**

From a study population of 140 children aged seven to 10 years, a sample of 31 children with flat feet was identified by screening with the FPI-6. Basic anthropometric measures were compared between subjects with and without flat feet as designated.

**Results:**

The results of this study, in contrast to many others, question the association of flat feet and heavy children. A significant relationship between foot posture and weight (FPI (L) r = -0.186 (p < 0.05), FPI(R) r = -0.194 (p < 0.05), waist girth (FPI (L) r = -0.213 (p < 0.05), FPI(R) r = -0.228 (p < 0.01) and BMI (FPI (L) r = -0.243 (p < 0.01), FPI(R) r = -0.263 (p < 0.01) was identified, but was both weak and inverse.

**Conclusions:**

This study presents results which conflict with those of many previous investigations addressing the relationship between children's weight and foot posture. In contrast to previous studies, the implication of these results is that heavy children have less flat feet. Further investigation is warranted using a standardized approach to assessment and a larger sample of children to test this apparent contradiction.

## Background

Over the last decade, the incidence of childhood obesity has increased across the globe [1,2]. The significance of overweight and obesity in children and relationship to foot morphology, specifically that of "flat feet", has been investigated by numerous authors [2-7]. Obesity is associated with many orthopaedic problems, yet few studies have closely examined the specific influence of excess body mass in children. Typical lower limb complications cited as possibly associated with obesity include: musculoskeletal pain, fractures, increased tibial/genu varum (Blount's disease), slipped capital femoral epiphysis, and a flat foot posture [2]. The paediatric flat foot is a controversial topic within the general community, medical and allied health fields, and has been debated and disputed for decades [8-19]. Despite this, there are huge gaps in our knowledge about flatfoot, as identified by a review [20].

The definition of flat foot is not standardized, nevertheless, there is general consensus that the height of the medial longitudinal arch is the principal parameter to be observed and measured [2,21]. The presence of flat footed posture has long been described as a foot abnormality often associated with pain and poor function. For this reason, many parents are naturally anxious to obtain prophylactic advice and treatment if they suspect that their child may suffer from this condition.

Overweight and obesity are well recognized as health problems and have been internationally standardized for children [1]. Previous investigation has found that both overweight and obesity were associated with flat foot posture in 835 children aged three to six years with flat foot found in 51% overweight children, 62% of obese children, and 42% of children of normal weight [22]. A German study used a scanner to investigate the influence of body mass on the development of a child's foot in 1450 boys and 1437 girls aged 2-14 years. This study identified five types of feet: *flat*, *robust*, *slender*, *short *and *long*. *Flat *and *robust feet *were more common in overweight children, whereas underweight children showed more *slender *and *long feet *[23]. Similarly in a study of 1024 Taiwanese children aged five to 13 years, there was significant difference in the prevalence of flatfoot between normal-weight (27%), overweight (31%), and obese (56%) children [24]. Another Taiwanese study sampled 2,083 children, between 7 and 12 years of age, determining the presence of flatfoot from footprints. Using this method, 59% of children were documented with flatfoot. The incidences of flatfoot were: 67% of males, 49% of females, and 75%, 65%, 57%, and 48% of obese, overweight, normal weight, and underweight children, respectively. A preponderance of flatfoot was observed among eight year olds, with males twice as likely to have flatfoot as females. Children who were obese or overweight were found to be 2.66 and 1.39 times more likely to have flatfoot than those of average weight [25]. Similar findings have been found in previous studies conducted on overweight and obese Australian children [3,5].

Clinicians often disagree about the management of flatfeet [26,27], partly because there is no standard approach to assessment or classification. This study investigated the relationship between flat foot posture, as rated by the FPI-6 method, and body weight and related anthropometric measurements, in a sample of Australian school children aged seven to ten years.

## Methods

Ethical approval was obtained from the Human Research and Ethics committee at the University of South Australia. Two primary schools in Port Pirie were approached and consented to being involved in the study. Consent forms were returned from the parents of 140 children, aged between seven and 10 years. Gender distribution for the study population consisted of 68 males and 72 females. Demographic data was collected from the returned consent forms as was inclusion (age)/exclusion (no history of foot surgery or congenital disorders) criteria.

The 140 children were assessed by one examiner using the Foot Posture Index (FPI-6) to establish basic static foot posture [28,29]. The FPI-6 is a scaled instrument widely used to classify foot posture along a 12 point continuum from pronated-normal-supinated. Scores which are positive are pronated, diverging from zero in the direction of a flat foot, where as negative scores indicate a supinated foot posture. Normative data sets show that FPI-6 scores of six and above are indicative of foot types more pronated beyond the mean value/age than the normal range for childhood [30]. The reliability of this examiner's use of the foot posture index has been previously established [31]. Thirty-one children were found to have a FPI-6 raw score of ≥ 6 for both feet [32] and were deemed to have flat feet [30].

The following general body anthropometric measurements for each child were made and recorded by an additional research assistant: height, weight and waist girth. Height was measured using a calibrated height gauge, weight using digital read-out scales and waist girth was measured using a standard tape measure [1]. All measures were recorded against each child's allocated identity (ID) code. All measures were performed with children dressed, but with shoes and socks removed.

### Data analysis

The recorded assessments yielded both categorical and continuous data. Descriptive statistics (mean, standard deviation, minimum, maximum, frequencies) were used to examine the basic anthropometrical characteristics of the study population. Parametric statistical correlations (Pearson's *r*) were applied to continuous data, and scatter plots were used to explore and illustrate relationships between parameters. An independent samples t- test was used compare group means for BMI, with Levene's test for equality of variance.

Data were entered and all analyses were performed using constructed data sets in SPSS version 15 (SPSS Science, Chicago, Illinois) and Microsoft Excel 2000 (Microsoft Inc, Redmond, Washington) software packages.

## Results

Anthropometric data for the whole sample population (N = 140), the non-flat foot group (n = 109) and for the flat foot group (n = 31) are shown in Table 1. From this, it can be seen that the average basic measures of anthropometry were largely independent of foot posture across the three groups as defined, where the anthropometric means did not differ greatly. An independent samples t-test found significant difference between the flat foot group (mean 17.28, SD 2.59) and the non-flat foot group (mean 18.74, SD 3.63) BMI's. Levene's test for equality of variances was not significant (F = 2.07, Sig = 0.15), hence assuming equal variances, two-tailed significance p = 0.017 (95% CI -2.653 to -0.268).

**Table 1:** Anthropometric descriptive statistics for the population sample (N = 140), the flatfoot group (n = 31) and the non-flatfoot group (n = 109).

	Age (years)	Height (cm)	Weight (kg)	BMI **(kg/m**^**2**^**)**	Waist (cm)	FPI-6 total Left	FPI-6 total Right
Mean							
All	8.71	132.85	32.77	18.30	67.36	4.12	3.74
Flat feet	8.58	133.48	30.87	17.26	64.87	6.61	6.68
Non- flat feet	8.75	132.48	33.05	18.49	67.67	3.58	3.15

Std. deviation							
All	0.91	8.85	9.93	3.39	9.95	2.23	2.34
Flat feet	0.92	7.10	5.89	2.55	7.26	0.80	0.70
Non- flat feet	0.91	9.33	10.53	3.49	10.41	1.98	2.03

Range							
All	3.00	55.00	85.50	24.16	69.00	11.00	11.00
Flat feet	3.00	29.00	20.70	9.67	27.00	3.00	2.00
Non- flat feet	3.00	55.00	85.50	24.16	69.00	8.00	9.00

Minimum							
All	7.00	110.00	17.80	13.78	53.00	-2.00	-3.00
Flat feet	7.00	116.00	21.70	14.16	55.00	6.00	8.00
Non- flat feet	7.00	110.00	17.80	13.78	53.00	-2.00	-3.00

Maximum							
All	10.00	165.00	103.30	37.94	122.00	9.00	8.00
Flat feet	10.00	145.00	42.40	23.83	82.00	9.00	8.00
Non- flat feet	10.00	165.00	103.30	37.94	122.00	8.00	9.00

**Figure 1:**
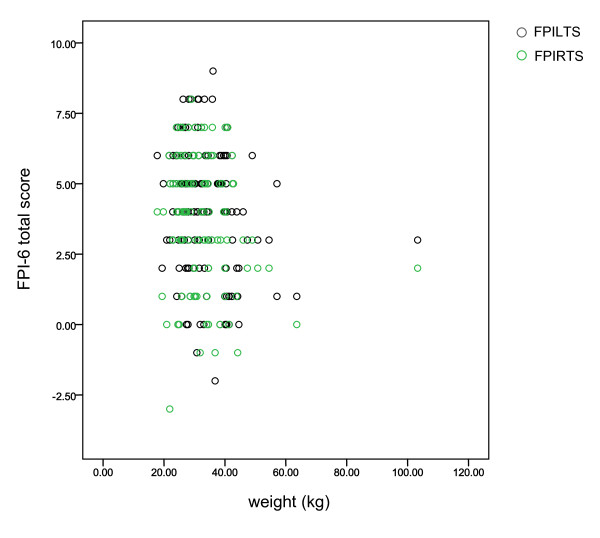
**This scatter plot of subjects' foot posture and weight revealed the obvious outlying position of one subject**. (FPILTS: FPI-6 left foot total score; FPIRTS: FPI-6 right foot total score).

During data collection and subsequent analysis, it was obvious to the examiner and assistant that one subject's anthropometry was clearly greater than all others. The scatter plot in Figure 1 reveals this relative outlier in terms of weight (this was also evident for height, waist and BMI). In order to assess the effect of this atypical subject, comparative descriptive statistics were examined for all subjects (N = 140) versus all subjects less the outlier (N = 139) (Table 2). The descriptive statistics mean values for height, weight, BMI and waist were very similar with (N = 140) and without (N = 139) the outlying subject, whilst as expected, standard deviations were greater with the outlier included.

**Table 2:** The effect of the outlier (depicted in Figure 1) was investigated for potential to skew the data.

	Height (cm)	Weight (kg)	**BMI (kg/m**^**2**^**)**	Waist (cm)
N = 140	132.85 (8.85)	32.77 (9.93)	18.30 (3.39)	67.36 (9.95)
range	110 - 165	17 - 103	13 - 37	53 - 122
				
N = 139	132.62 (8.45)	32.26 (7.93)	18.13 (2.95)	66.96 (8.82)
	110 - 155	17 - 63	13 - 26	53 - 96

The World Health Organisation reference charts for children's BMI across age groups indicate that normal BMI for children aged seven to 10 years are 15.5 - 16.5 kg/m^2 ^in boys and 15.5 - 17.0 kg/m^2 ^in girls. The mean BMI for the 140 children in this study was 18.3 ± 3.4 kg/m^2^, with a wide range of BMI results: 13.7 - 37.9 kg/m^2^. The BMI cut-off points of the International Obesity Task Force (IOTF) were used to delineate overweight children per year of age [33]. As depicted in Table 3, 55/140 children were classified as overweight using the IOTF criteria. Flat feet were found in 31/140 children. Only 5 children with flatfeet were also overweight. (Given the primary purpose of exploring the relationship between foot posture and BMI, distinction was not made between overweight and obesity).

**Table 3:** Children, according to age groups, foot posture and BMI cut-off points.

Age (years)	No. children (-/140 total (%))	No. children with flat feet	No. children without flat feet	BMI - cut off points/age [International Obesity Task Force]	No. of overweight children vs foot posture (-/mean FPI-6 L: R)	No. of overweight children with flat feet
						
7	11 (7.4)	3	8	18	3/6.3: 5.0	1
						
8	50 (33.8)	13	37	18.5	10/4.4: 3.7	1
						
9	47 (31.8)	9	38	19	21/3.1: 2.6	3
						
10	32 (21.6)	6	26	20	11/2.6: 2.2	0
						
Total no. children	140	31	109	-	55	5

Using the international cut-off points for overweight (BMI 25 kg/m^2^) 55/140 children were found to be overweight. Only five of the overweight children also had flat feet (FPI-6 greater or equal to 6 points on both left and right feet).

As shown in Table 4, there was significant and strong correlation between waist girth and weight (r = 0.938; p < 0.01), height (r = 0.664; p < 0.01) and BMI (r = 0.912; p < 0.01). Correlations between waist girth and foot posture (FPI (L) r = -0.213 (p < 0.05), FPI(R) r = -0.228 (p < 0.01), BMI and foot posture (FPI (L) r = -0.243 (p < 0.01), FPI(R) r = -0.263 (p < 0.01), weight and foot posture (FPI (L) r = -0.186 (p < 0.05), FPI(R) r = -0.194 (p < 0.05) were also significant, but weaker and inverse. Correlation between foot posture and height was not significant (p < 0.05).

**Table 4:** Waist girth correlated significantly with weight (r = 0.938; p < 0.01) and also height (r = 0.664; p < 0.01). Waist girth and foot posture correlations were weak and inverse viz. FPI (L) r = -0.213 (p < 0.05), FPI(R) r = -0.228 (p < 0.01).

		FPILTS	Height	Weight	BMI	Waist	FPIRT
**FPILTS**	Pearson Correlation	1	-.037	-.186(*)	-.243(**)	-.213(*)	.759(**
	
	Sig. (2-tailed)		.665	.028	.004	.011	.000

**Height**	Pearson Correlation	-.037	1	.759(**)	.458(**)	.664(**)	-.017
	
	Sig. (2-tailed)	.665		.000	.000	.000	.844

**Weight**	Pearson Correlation	-.186(*)	.759(**)	1	.909(**)	.938(**)	-.194(*)
	
	Sig. (2-tailed)	.028	.000		.000	.000	.021

**BMI**	Pearson Correlation	-.243(**)	.458(**)	.909(**)	1	.912(**)	-.263(**)
	
	Sig. (2-tailed)	.004	.000	.000		.000	.002

**Waist**	Pearson Correlation	-.213(*)	.664(**)	.938(**)	.912(**)	1	-.228(**)
	
	Sig. (2-tailed)	.011	.000	.000	.000		.007

**FPIRTS**	Pearson Correlation	.759(**)	-.017	-.194(*)	-.263(**)	-.228(**)	1
	
	Sig. (2-tailed)	.000	.844	.021	.002	.007	

* Correlation is significant at the 0.05 level (2-tailed).

** Correlation is significant at the 0.01 level (2-tailed).

(FPILTS: FPI-6 left foot total score; FPIRTS: FPI-6 right foot total score)

**Figure 2:**
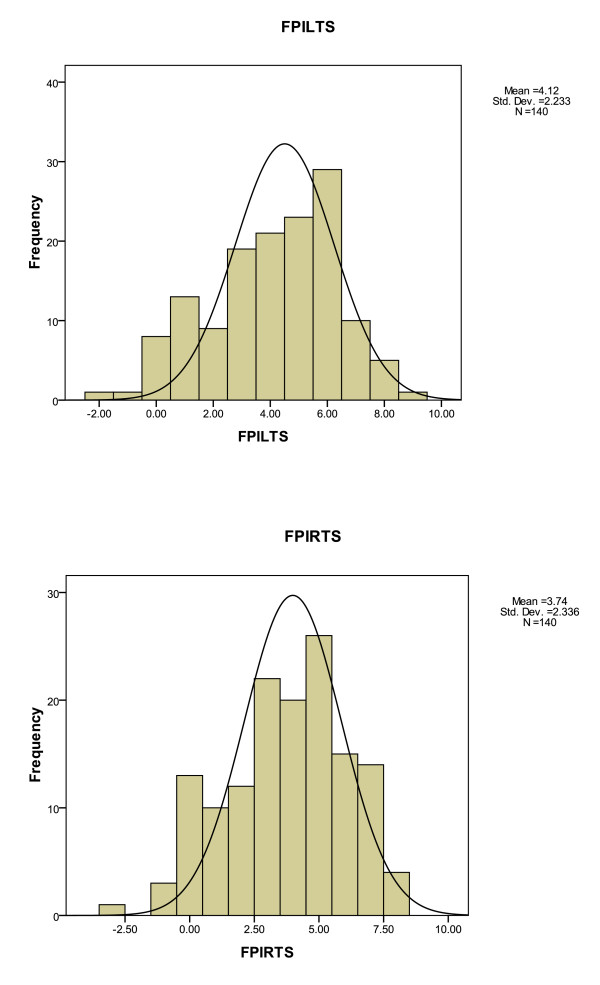
**The FPI-6 total scores for both left and right feet of the study population (N = 140), children aged seven to 10 years**.

The foot posture histograms for the study population (N = 140) (Figure 2) showed normal curve distribution for both left and right FPI-6 total scores. The FPI-6 left foot total score averaged 4.12 (± 2.2) and the FPI-6 right foot total score averaged 3.74 (± 2.3).

## Discussion

The anthropometry results in this study are notable for three main findings. Firstly, there was an overall lack of significant difference in basic anthropometrical attributes found between the flat foot versus the non-flat foot groups. Despite the significant difference in BMI between the flat foot and non-flat foot groups, the disparity in group sizes and the relatively small sample size of this study must be appreciated. However this study did not find the previously postulated/found result, whereby heavier children (i.e. increased body weight) had flatter feet [5,6,22-25].

Secondly, the measure of waist girth, commonly used to assess body visceral fat and predictive of secondary increased health risks (e.g. blood pressure, blood lipids, metabolic syndrome) [34] correlated well with both weight and height (and therefore BMI), which is unsurprising in that taller, heavier children are seen to have greater waist circumference. In comparison to the cut-off values in waist circumference for 90^th ^percentile for children in the US [35], where the average waist circumference across genders in children aged seven to 10 years was 74.4 cm (range 68.4 - 80.8 cm), the average waist circumference in our study population was 67.4 cm (range 53 - 122 cm); approximately ten percent less. Of greater interest perhaps, was the finding that the measure of waist girth was correlated (if weakly) with foot posture, yet inversely, whereby 'fatter' waists were related to *less *flat feet. This contrasts to the work of many previous authors including Pfeiffer who, in a larger study than this, found correlation between flat feet and weight/obesity in younger children [22]. This finding is however, supported by previously reported findings in younger children with leg pain (defined as 'growing pains'), which found that children who had growing pains were on average 5% heavier, but had less flat feet [36].

Thirdly, the FPI-6 scores indicate that a broad range of foot types i.e. supinated to pronated, were encountered within this study group, which is important for the external validity of these findings. The mean FPI-6 scores for the non-flat foot group of the study (n = 109) indicate that the average foot posture is mildly pronated in children aged seven to ten years, which supports the recently compiled normative values for the foot posture index [30].

This study examined 140 children aged seven to ten years, hence derives results from considerably fewer subjects than other investigations which have ranged from study populations of 835 to 2887 subjects [22-25]. The narrower age range of four years, delineates it from the larger studies and compares it with another similar study of 200 children, aged nine to 12 years [6]. Similar to the results of all other studies, this latter study also found positive relationship between a flatter foot posture and increased body weight. The results presented here are clearly dissonant to all previously published research. It is pertinent to remember that the largest studies have been performed in Taiwanese and German children, hence a different ethnicity profile. Studies which have investigated the relationship between body mass and foot posture are shown in Table 5.

**Table 5:** Study parameters of the investigations into paediatric foot posture and body mass, show that footprint measures have dominated foot posture assessment.

Year of publication	First author, country	Age of children (years)	Sample size (n)	Method of foot posture assessment	Flat feet related to increased body mass
2001	Dowling, Australia	8 - 9	26	Footprints, pressure mat	Yes
2006	Pfeiffer, Austria	3 - 6	835	Scanner, rearfoot angle	Yes
2006	Mickle, Australia	4 - 5	38	Footprints, ultrasound measure of heel fat pad	Yes
2007	Morrison, UK	9 - 12	200	Foot length/width, Navicular height	Yes
2008	Mauch, Germany	2 - 14	2887	Scanner	Yes
2009	Chen, Taiwan	5 - 13	1024	Footprints, 3D scan	Yes
2010	Chang, Taiwan	7 - 12	2083	Footprints	Yes
2011	Evans, Australia	7 - 10	140	FPI-6	No

The methods of identifying and classifying foot posture vary greatly between the studies examining this area. Many studies have used foot print measures [2,3,5,25], where in essence, greater surface area is related to lower medial longitudinal arch height. The validity of this widely used assumption remains unfounded however; it is possible that the greater surface area of the foot print is just soft tissue expansion and spread, rather than overt lowering of the medial osseous foot arch per se [7]. Other studies have looked at foot length, width and navicular height [6] or foot x-rays [2,21] to assess foot posture. This study employed the FPI-6 to rate subject's foot posture, an observational scale, for which normative values exist [30]. The data set of 1648 individual observations of foot posture (which was used to develop the FPI-6 normative values) in children, adults and older people, found no relationship between foot posture and BMI [30]. It is possible that the use of different foot posture measures may account for some of the discord between the present study's findings and those of other investigators.

## Conclusion

The findings of this study are at odds with many other similar investigations, in that not only did it did not find a positive relationship between increased body weight and flatter foot posture, it found the inverse. The sample size, subject ethnicity and assessment method of foot posture may be relevant contributors to this clear disparity, but this warrants further inquiry. Other unidentified variables may also be proponents of altered foot posture in children. A standardized and ideally a validated approach to the assessment of children's foot posture and its relationship to fundamental anthropometry is required to clarify whether any concern about (in particular) children's weight and foot posture is duly warranted.

## Competing interests

The authors declare that they have no competing interests.

## Authors' information

Angela M Evans is a Senior Research Fellow (adjunct) at the School of Health Science, Division of Health Science, University of South Australia.

## References

[B1] Cole TJ Bellizzi MC Flegal KM Dietz WH Establishing a standard definition for child overweight and obesity worldwide: international survey BMJ 2000 320 1 6 10.1136/bmj.320.7226.1 10797032 PMC27365

[B2] Villarroya MA Esquivel JM Tomás C Moreno LA Buenafé A Bueno G Assessment of the medial longitudinal arch in children and adolescents with obesity: footprints and radiographic study Eur J Pediatr 2009 168 559 567 10.1007/s00431-008-0789-8 18751725

[B3] Dowling AM Steele JR Baur LA Does obesity influence foot structure and plantar pressure patterns in prepubescent children? Int J Obes 2001 25 845 852 10.1038/sj.ijo.0801598 11439299

[B4] El O Akcali O Kosay C Kaner B Arslan Y Sagol E Flexible flatfoot and related factors in primary school children: a report of a screening study Rheumatol Int 2006 26 1050 1053 10.1007/s00296-006-0128-1 16670858

[B5] Mickle KJ Steele JR Munro BJ The feet of overweight and obese young children: are they flat or fat? Obesity 2006 14 1949 1953 10.1038/oby.2006.227 17135610

[B6] Morrison SC Durward BR Watt GF Donaldosn MDC Anthropometric Foot Structure of Peripubescent Children with Excessive versusNormal Body Mass. A Cross-sectional Study J Am Podiatr Med Assoc 2007 97 366 370 17901340 10.7547/0970366

[B7] Onodera AN Saccoa ICN Morioka EH Souza PS deSa MR madio AC What is the best method for child longitudinal plantar arch assessment and when does arch maturation occur? Foot 2008 18 142 149 10.1016/j.foot.2008.03.003 20307428

[B8] Alakija W Prevalence of flat foot in school children in Benin City, Nigeria Trop Doct 1979 9 192 194 516145 10.1177/004947557900900425

[B9] Bordelon RL Hypermobile flatfoot in children. Comprehension, evaluation, and treatment Clin Orthop Relat Res 1983 Dec 7 14 6641070

[B10] D'Amico JC Developmental flatfoot Clin Podiatry 1984 1 535 546 6536407

[B11] Ferciot CF The etiology of developmental flatfoot Clin Orthop Relat Res 1972 85 7 10 5036934 10.1097/00003086-197206000-00003

[B12] Gervis WH Flat foot BMJ 1970 1 479 481 10.1136/bmj.1.5694.479 5435151 PMC1699486

[B13] McCarthy DJ The developmental anatomy of pes valgo planus Clin Podiatr Med Surg 1989 6 491 509 2665922

[B14] Miller GR The operative treatment of hypermobile flatfeet in the young child Clin Orthop Relat Res 1977 Jan-Feb 95 101 837625

[B15] Staheli LT Planovalgus foot deformity. Current status J Am Podiatr Med Assoc 1999 89 94 99 10063780 10.7547/87507315-89-2-94

[B16] Sullivan JA Pediatric flatfoot: evaluation and management J Am Acad Orthop Surg 1999 7 44 53 9916191 10.5435/00124635-199901000-00005

[B17] Suzuki N An electromyographic study of the role of muscles in arch support of the normal and flat foot Nagoya Med J 1972 17 57 79 5050758

[B18] Tareco JM Miller NH MacWilliams BA Michelson JD Defining flatfoot Foot Ankle Int 1999 20 456 460 10437930 10.1177/107110079902000711

[B19] Tax HR Flexible flatfoot in children J Am Podiatr Med Assoc 1977 67 616 619 10.7547/87507315-67-9-616 903565

[B20] Harris EJ The natural history and pathophysiology of flexible flatfoot Clin Podiatr Med Surg 2010 27 1 23 10.1016/j.cpm.2009.09.002 19963167

[B21] Kanatli U Yetkin H Cila E Footprint and Radiogeaphic Analysis of the feet J Pediatr Orthop 2001 21 225 228 11242255

[B22] Pfeiffer M Kotz R Ledl T Hauser G Sluga M Prevalence of flat foot in preschool-aged children Pediatrics 2006 118 634 639 10.1542/peds.2005-2126 16882817

[B23] Mauch M Grau S Krauss I Maiwald C Horstmann T Foot morphology of normal, underweight and overweight children Int J Obes (Lond) 2008 32 1068 1075 10.1038/ijo.2008.52 18414422

[B24] Chen JP Chung MJ Wang MJ Flatfoot prevalence and foot dimensions of 5- to 13-year-old children in taiwan Foot Ankle Int 2009 30 326 332 10.3113/FAI.2009.0326 19356357

[B25] Chang JH Wang SH Kuo CL Shen HC Hong YW Lin LC Prevalence of flexible flatfoot in Taiwanese school-aged children in relation to obesity, gender, and age Eur J Pediatr 2010 169 447 452 10.1007/s00431-009-1050-9 19756732

[B26] Bresnahan P The Flat-Footed Child - To Treat or Not to Treat. What is the Clinician to Do? J Am Podiatr Med Assoc 2009 99 178 19299359 10.7547/0980178

[B27] Evans AM The Flat-Footed Child - To Treat or Not to Treat. What is the Clinician to Do? J Am Podiatr Med Assoc 2009 99 179 10.7547/0980267 19448182

[B28] Keenan AM Redmond AC Horton M Conaghan PG Tennant A The Foot Posture Index: Rasch analysis of a novel, foot specific outcome measure Rheumatology 2006 45 i128 10.1016/j.apmr.2006.10.005 17207681

[B29] Redmond AC Crosbie J Ouvrier R Development and validation of a novel rating system for scoring foot posture: the Foot Posture Index Clin Biomech 2006 21 89 98 10.1016/j.clinbiomech.2005.08.002 16182419

[B30] Redmond AC Crane YZ Menz HB Normative values for the Foot Posture Index J Foot Ankle Res 2008 1 10.1186/1757-1146-1-6 PMC2553778 18822155

[B31] Evans AM Copper AW Scharfbillig RW Scutter SD Williams MT Reliability of the Foot Posture Index and Traditional Measures of Foot Position J Am Podiatr Med Assoc 2003 93 203 12756311 10.7547/87507315-93-3-203

[B32] Evans AM Scutter S Lang LMG Dansie BR 'Growing pains' in young children: A study of the profile, experiences and quality of life issues of four to six year old children with recurrent leg pain The Foot 2006 16 120 124 10.1016/j.foot.2006.02.006

[B33] Lobstein T Baur L Uauy R Obesity in children and young people: a crisis in public health Obes Rev 2004 5 4 85 10.1111/j.1467-789X.2004.00133.x 15096099

[B34] Lee S Bacha F Arslanian SA Waist circumference, blood pressure, and lipid components of the metabolic syndrome J Pediatr 2006 149 809 816 10.1016/j.jpeds.2006.08.075 17137898

[B35] Li C Ford ES Mokdad AH Cook S Recent Trends in Waist Circumference and Waist-Height Ratio Among US Children and Adolescents Pediatrics 2006 118 1390 1398 10.1542/peds.2006-1062 17079540

[B36] Evans AM Scutter S Are Foot Posture and Functional Health different in Children with Growing Pains? Pediatr Int 2007 49 991 996 10.1111/j.1442-200X.2007.02493.x 18045309

